# P-1115. Acinetobacter baumannii Outbreak in a Neonatal ICU: Only a Case-Control Study Could Identify the Nest

**DOI:** 10.1093/ofid/ofaf695.1310

**Published:** 2026-01-11

**Authors:** Adelino M Freire Junior, Rosilu F Barbosa, Polyana O Andrade, Bráulio R G M Couto

**Affiliations:** Unimed-BH, Belo Horizonte, Minas Gerais, Brazil; Unimed-BH, Belo Horizonte, Minas Gerais, Brazil; Unimed-BH, Belo Horizonte, Minas Gerais, Brazil; AMECI – Associação Mineira de Epidemiologia e Controle de Infecções, Belo Horizonte, Minas Gerais, Brazil

## Abstract

**Background:**

Carbapenem-resistant *Acinetobacter baumannii* (CRAB) infection in the Neonatal Intensive Care Unit (NICU) is a serious threat. Between Apr-Aug/2024, six CRAB bloodstream infections prompted aggressive outbreak control: strict hand hygiene, colonization screening (surfaces, neonates, staff), enhanced cleaning/disinfection, empirical antibiotic protocol review, and CVC bundle review (including PICC). Even after these measures, CRAB cases still increased to 14 cases in Sep/2024 (Figure 1). Here, we present the results of the case-control study performed to investigate the CRAB outbreak.

**Figure 1:**
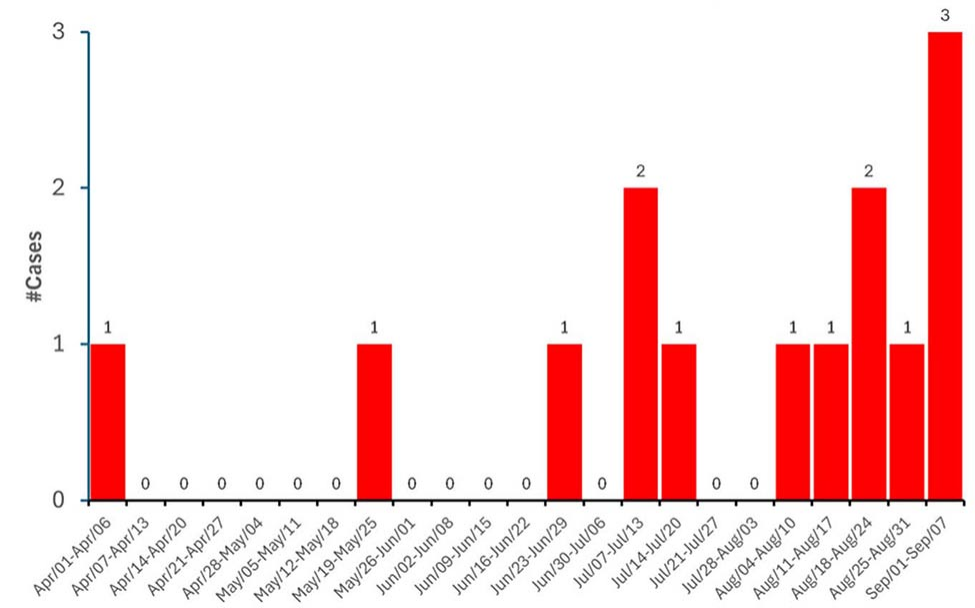
Epidemic curve of Carbapenem-resistant Acinetobacter baumannii (CRAB) infection in a Neonatal Intensive Care Unit (NICU). Epidemic curve of Carbapenem-resistant Acinetobacter baumannii (CRAB) infection in a Neonatal Intensive Care Unit (NICU): a total of 14 cases were identified between Apr-Sep/2024, indicating a significant increase by the end of September 2024.

**Table 1:**
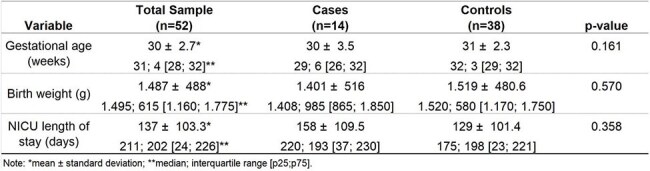
Univariate analysis of the quantitative variables. Univariate analysis of the quantitative variables: these factors were not associated with the CRAB outbreak.

**Methods:**

For each CRAB case we selected three controls, neonates without any infection or colonization, paired by birth weight. A total of 27 variables were collect, including birth conditions, comorbidities, presence of invasive procedures, surgery, use of antibiotics. Data were registered in a Google Sheet and analyzed by Epi Info.

**Table 2:**
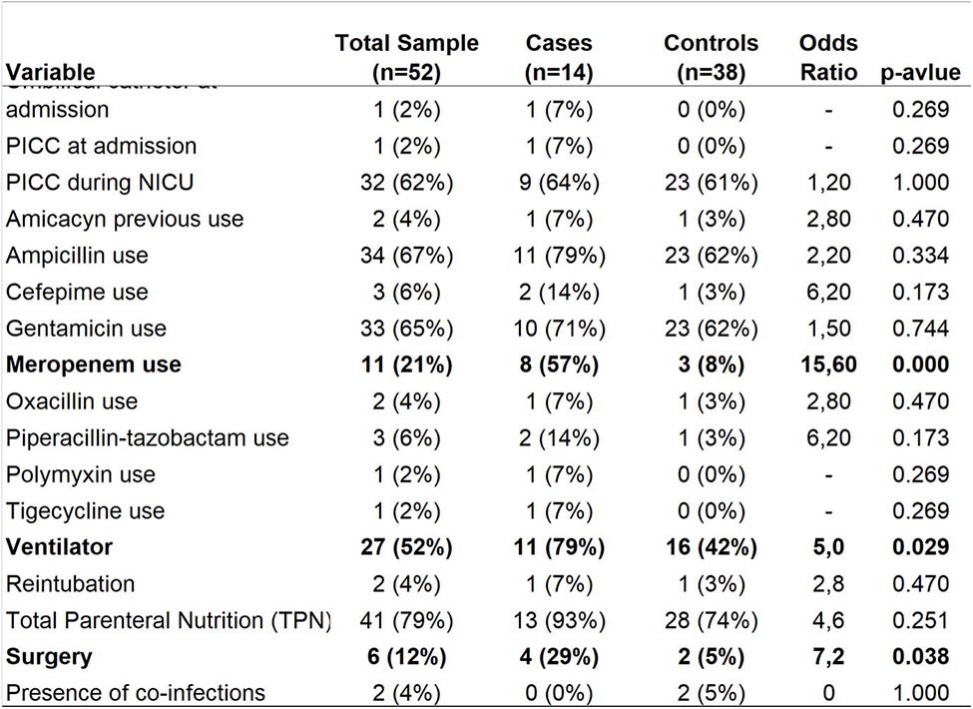
Univariate analysis of the categorical variables. Univariate analysis of the categorical variables: only meropenem use, surgery and ventilator were significantly associated with the CRAB outbreak.

**Figure 2:**
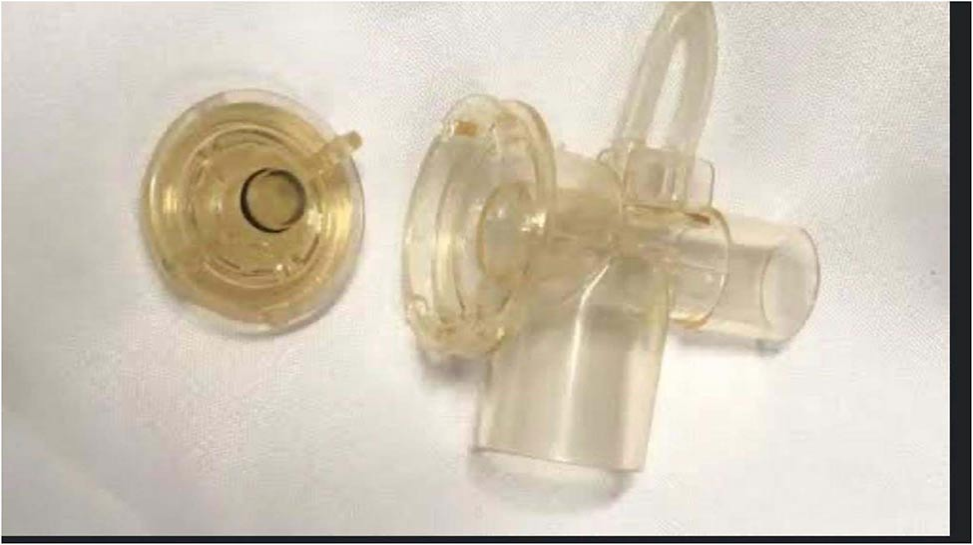
Investigations revealed that ventilator filters were positive for Acinetobacter baumannii. Investigations revealed that ventilator filters were positive for Acinetobacter baumannii.

**Results:**

A total of 14 cases and 38 controls were analyzed (some cases had fewer than three controls). Due to the pairing method, neither gestational age nor birth weight differed significantly between cases and controls (Table 1). The univariate analysis of categorical variables identified that meropenem use, surgery, and ventilator use were significantly associated with the CRAB outbreak. An aggressive investigation into these three factors was immediately started. No specific results were found initially. Surgical procedures were evaluated by infection control personnel. A thorough microbiological investigation was conducted on the ventilator system and its components, including cleaning and disinfection procedures. Subsequently, the likely source, or "nest," was identified: *Acinetobacter baumannii* was found in the ventilator filters.

**Conclusion:**

Enforcing strict hand hygiene, colonization screening, enhanced cleaning/disinfection, empirical antibiotic protocol review, and CVC bundle review were insufficient to control the CRAB outbreak. Only a qualitative-quantitative analysis, associating the results of a case-control study with qualitative investigation, was capable of identifying the CRAB reservoir and stopping the outbreak. After correcting the ventilator filter disinfection procedures, no further CRAB cases occurred.

**Disclosures:**

All Authors: No reported disclosures

